# Phosphorylation of the *Drosophila melanogaster* RNA–Binding Protein HOW by MAPK/ERK Enhances Its Dimerization and Activity

**DOI:** 10.1371/journal.pgen.1002632

**Published:** 2012-03-29

**Authors:** Ronit Nir, Rona Grossman, Ze'ev Paroush, Talila Volk

**Affiliations:** 1 Department of Molecular Genetics, Weizmann Institute of Science, Rehovot, Israel; 2 Department of Developmental Biology and Cancer Research, Institute for Medical Research Israel-Canada (IMRIC), Faculty of Medicine, The Hebrew University, Jerusalem, Israel; Harvard Medical School, Howard Hughes Medical Institute, United States of America

## Abstract

*Drosophila melanogaster* Held Out Wings (HOW) is a conserved RNA–binding protein (RBP) belonging to the STAR family, whose closest mammalian ortholog Quaking (QKI) has been implicated in embryonic development and nervous system myelination. The HOW RBP modulates a variety of developmental processes by controlling mRNA levels and the splicing profile of multiple key regulatory genes; however, mechanisms regulating its activity in tissues have yet to be elucidated. Here, we link receptor tyrosine kinase (RTK) signaling to the regulation of QKI subfamily of STAR proteins, by showing that HOW undergoes phosphorylation by MAPK/ERK. Importantly, we show that this modification facilitates HOW dimerization and potentiates its ability to bind RNA and regulate its levels. Employing an antibody that specifically recognizes phosphorylated HOW, we show that HOW is phosphorylated in embryonic muscles and heart cardioblasts *in vivo*, thus documenting for the first time Serine/Threonine (Ser/Thr) phosphorylation of a STAR protein in the context of an intact organism. We also identify the *sallimus/D-titin* (*sls*) gene as a novel muscle target of HOW–mediated negative regulation and further show that this regulation is phosphorylation-dependent, underscoring the physiological relevance of this modification. Importantly, we demonstrate that HOW Thr phosphorylation is reduced following muscle-specific knock down of *Drosophila* MAPK *rolled* and that, correspondingly, Sls is elevated in these muscles, similarly to the HOW RNAi effect. Taken together, our results provide a coherent mechanism of differential HOW activation; MAPK/ERK-dependent phosphorylation of HOW promotes the formation of HOW dimers and thus enhances its activity in controlling mRNA levels of key muscle-specific genes. Hence, our findings bridge between MAPK/ERK signaling and RNA regulation in developing muscles.

## Author Summary

Somatic muscles are huge cells that feature highly organized sarcomeric architecture, whose formation and maintenance are not fully understood. Multiple signals play a role in these cells, including the highly conserved MAPK/ERK pathway, which often serves as a cue for cellular proliferation or differentiation. In this study, we reveal a role for MAPK/ERK signaling in the regulation of multiple muscle genes at the level of mRNA, through the control of the activity of the *Drosophila* RNA–binding protein Held Out wings (HOW). Specifically, we show that HOW undergoes phosphorylation by MAPK/ERK, which increases its ability to form dimers and enhances its RNA–binding capacity. We further demonstrate that HOW is phosphorylated in embryonic and larval muscles by MAPK *in vivo* and that this event is important for its ability to regulate the levels of a giant sarcomeric gene homologous to vertebrate *titin*, thus contributing to the maintenance of muscle sarcomeric architecture. Importantly, HOW is a close homolog of mammalian Quaking, an essential protein in embryonic development and nervous system myelination, a reduction of which is correlated with Schizophrenia. Thus, our results raise the possibility that MAPK/ERK phosphorylation similarly regulates RNA profiles in other tissues controlled by proteins of the conserved Quaking family.

## Introduction

Regulation of gene expression at the level of RNA is often mediated through the activities of RNA-binding proteins (RBPs), which control different aspects of RNA metabolism of target genes [1], [2]. *Drosophila melanogaster* Held Out Wings (HOW) is an RBP that belongs to a family of evolutionarily conserved “Signal Transduction and Activation of RNA” (STAR) proteins [3]. STAR family members control a wide range of tissue differentiation processes. For example, in mammals, Sam68 controls spermatogenesis [4], and Quaking (QKI) regulates myelination by Schwann cells and oligodendrocytes [5], [6], [7] as well as muscle fiber maturation in Zebrafish [8]. In *C. elegans*, the STAR protein GLD-1 promotes germ cell differentiation [9], while ASD-2 is required for alternative splicing [10].

HOW, the *Drosophila* protein orthologous to mammalian QKI, is highly expressed in muscles, tendons [11], [12], [13], [14] and glial cells [15], [16], where it plays an essential role during development by controlling the mRNA levels of an array of target genes [17]. HOW performs various activities on its target RNAs: it facilitates the alternative splicing of *stripe A*, a transcription factor essential for tendon cell maturation [18], and mediates specific splicing of the septate junction constituent, *nrxIV*, thereby controlling glial cell maturation [15]. It also functions by reducing mRNA levels of various targets. For example, during gastrulation, HOW-dependent downregulation of *cdc25/string*, a cell cycle promoting phosphatase, is essential to inhibit cell division in invaginating mesodermal cells [19].

Structurally, HOW contains a single maxi-KH RNA binding motif that is flanked by two additional conserved domains, QUA1 and QUA2 [14]. While the QUA2 motif, located C terminally to the KH domain, takes part in RNA-binding and contributes to the specificity of RNA recognition [20], [21], [22], the QUA1 motif, located N terminally to the KH domain, was shown to mediate protein dimerization in GLD-1, Sam68 and QKI [23], [24], [25]. Notably, despite the fact that this domain is not essential for RNA-binding, its deletion in GLD-1 nevertheless reduces the affinity of the protein to the RNA binding motif TGE (Tra-2 and GLI response element), by about ten-fold [26], suggesting that dimerization of STAR proteins might enhance their affinity to RNA. To date, it is not clear what regulates the degree of STAR protein dimer formation.

Consistent with their expression in a wide range of tissues during development, the activity of STAR proteins is highly regulated at distinct post-translational levels, including phosphorylation by various kinases (reviewed in [27]). These modifications likely impinge on the activity, subcellular distribution, or the formation of protein complexes of specific STAR proteins. Phosphorylation of STAR proteins could couple regulation of RNA metabolism with distinct signaling cascades operating in a spatial and temporal restricted manner.

While Tyrosine phosphorylation of the C terminal regions of both Sam68 [28] and QKI [29] has been established, Serine/Threonine (Ser/Thr) phosphorylation has yet to be demonstrated with respect to the QKI-subfamily of proteins. A more evolutionarily distant STAR protein,Sam68, was shown to be Ser/Thr phosphorylated, both by Cdc2 [30] and by ERK1/2 in various culture lines, promoting differential activities. Specifically, Sam68 phosphorylation by ERK1/2 in a lymphoma cell line enhances its ability to regulate alternative splicing [31], while in mouse spermatocytes and in HEK293 cells it induces cytoplasmic accumulation correlated with its association with polyribosomes [4], [32]. We therefore postulated that ERK-dependent phosphorylation of STAR proteins might affect their activity in a tissue-specific manner.

In the present manuscript, we show that the *Drosophila* STAR protein HOW is phosphorylated on conserved Thr residues. Importantly, we demonstrate that in embryos *in vivo*, a particular HOW isoform is phosphorylated on Thr, in a tissue-specific manner. Moreover, we identify the major Z-disc gene product *sallimus* (*kettin/D-titin*, *sls* [33], [34]) as a specific target for HOW in muscle cells and show that *sls* regulation is dependent on MAPK phosphorylation of HOW. Mechanistically, we demonstrate that HOW phosphorylation is essential for its efficient homodimerization and RNA binding capability. Taken together, our results reveal a molecular mechanism linking muscle-specific MAPK-dependent phosphorylation of HOW to its ability to homodimerize, bind its targets and regulate them, and thereby contribute to muscle sarcomerization.

## Results

### HOW contains conserved consensus sites for MAPK/ERK phosphorylation

**Figure 1:**
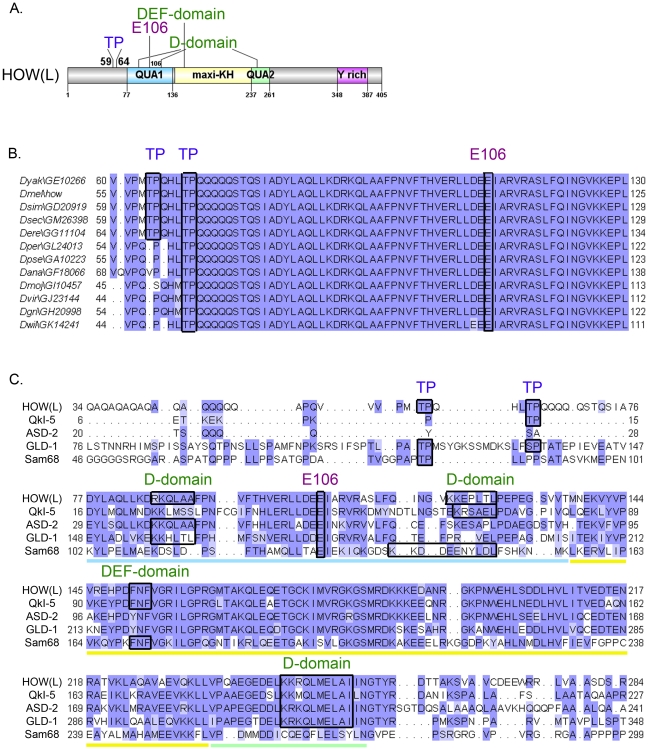
HOW possesses conserved MAPK/ERK consensus phospho-acceptor sites and docking domains. A. A scheme of HOW(L) protein domains. The locations of the TP sites at positions 59 and 64, the Glutamic acid at position 106 required for homodimerization, and the four potential MAPK/ERK docking sites are indicated. The STAR/GSG domain is indicated by colored squares: The QUA1 domain (light blue), the maxi-KH domain (yellow), and the QUA2 domain (green). Also indicated is the Tyrosine rich area in the C-terminus (pink). B. Alignment of HOW(L) amino acid sequences from 12 *Drosophila* species. While T64 is conserved throughout all the species, T59 is only conserved in five of them (conserved residues are demarcated by black frames). E106 is also fully conserved, as are the putative docking sites (not shown). C. Alignment of the HOW(L) protein with the mammalian proteins QKI-5 and Sam68, and the nematode homologs, GLD-1 and ASD-2. The Qua1 domain is underlined in light blue, the maxi-KH domain in yellow, and the Qua2 in green. Note the conserved TP sites, E106 and various docking sites, demarcated by frames. Both the DEF domain and the second D-domain (at a.a. 121) are conserved in the mammalian proteins QKI and Sam68, whereas the first (at a.a. 85) and third (at a.a. 245) D-domains are conserved in C. elegans proteins (the latter is also conserved in QKI).

Examination of HOW revealed two putative MAPK/ERK consensus sites, comprised of Thr followed by Proline (Pro) (TP) at residues 59 and 64 (Figure 1A). These could also serve as putative sites for other Ser/Thr kinases such as Cyclin Dependent Kinases (CDKs). Importantly, T64 is conserved throughout HOW(L) sequences in all annotated *Drosophila* species, while T59 is found only in closely related species (Figure 1B).

In addition to these conserved phosphorylation sites, HOW also contains putative MAPK docking sites. These “D” [35] or “DEF” [36] domains frequently mediate high-affinity interactions between MAPK and its substrates, allowing efficient phosphorylation of the substrate [37]. HOW harbors three such potential D-domain motifs, **^85^RK**Q**L**A**A**, ^121^ **KK**EP**L**T**L**, and ^245^ **KKR**QLME**L**A**I**, as well as a single DEF domain motif, ^151^ **FNF**. All four motifs are fully conserved throughout *Drosophila* species, and partially conserved in other STAR proteins (Figure 1C). The presence of these domains, together with the occurrence of potential phosphorylation sites, led us to test the possibility that HOW is phosphorylated by MAPK/ERK.

### HOW is phosphorylated by MAPK/ERK *in vitro*

To start investigating phosphorylation of HOW by MAPK/ERK, we generated a HOW construct in which the putative phosphoacceptor sites T59 and T64 were mutated to Alanine (HOW^TTAA^), rendering it non-phosphorylatable.

**Figure 2:**
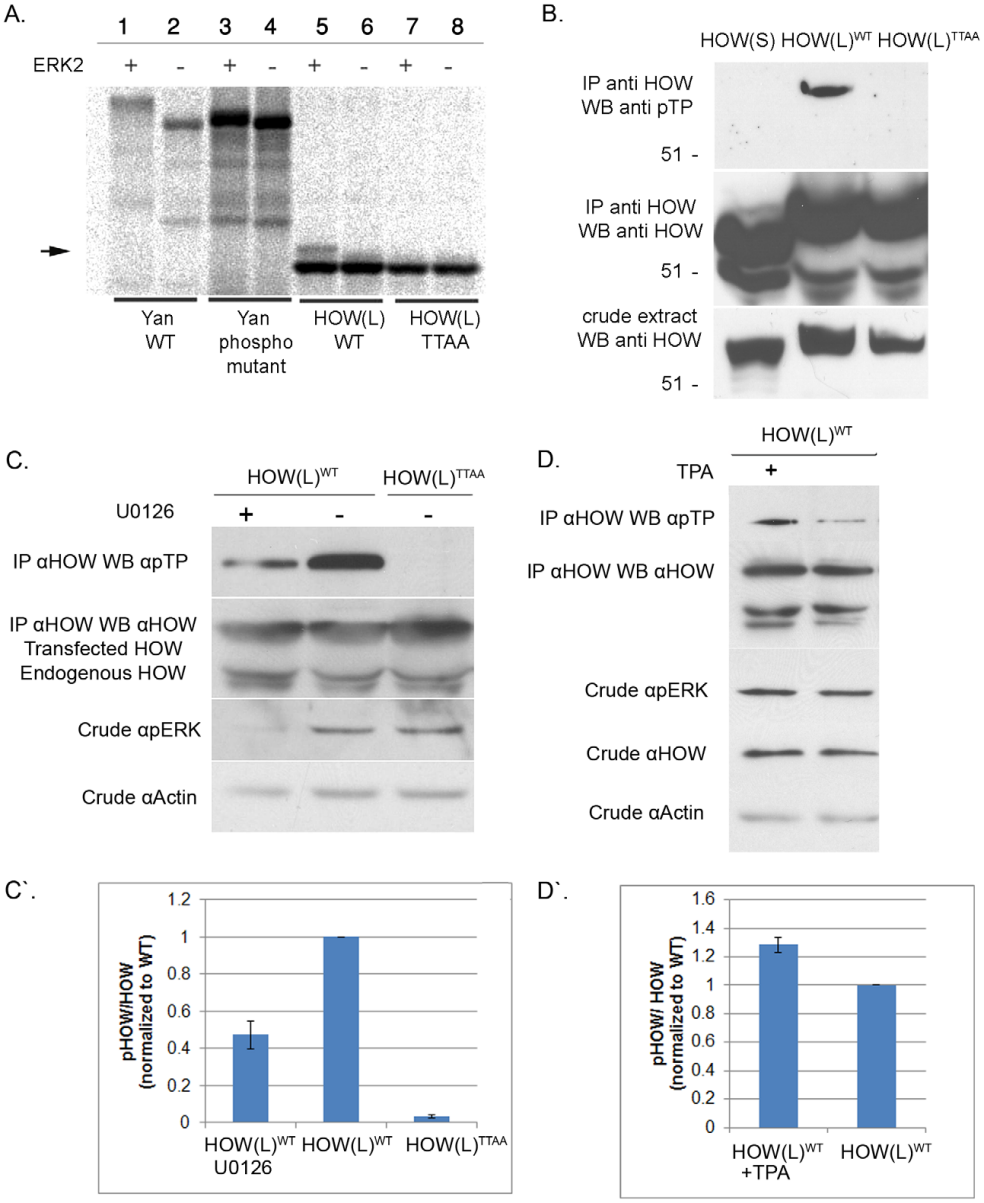
HOW(L) is phosphorylated *in vitro* and in S2R+ cells by MAPK/ERK on one or more TP sites. A. ERK2 phosphorylates HOW *in vitro*. HOW(L)^WT^ and HOW(L)^TTAA^ (phospho mutant) constructs were *in vitro* translated (using S^35^ labeling) and incubated with activated ERK2. The reaction was resolved on an SDS gel. The left part of the panel (lanes 1–4) shows phosphorylation of the Yan (Aop) protein as a control. HOW(L)^WT^ protein, but not the HOW(L)^TTAA^ mutant (lanes 7–8) exhibits a molecular weight shift of a fraction of the protein when incubated with the kinase (lane 5) relative to HOW without the kinase (lane 6). B. HOW(L), but not HOW(S), is phosphorylated in S2R+ cells. HOW proteins were immunoprecipitated using an anti-HOW antibody and reacted with anti-pTP (top panel) and anti-HOW (middle panel) antibodies. HOW proteins in the crude extract are shown in the bottom panel. From the left: HOW(S)^WT^, HOW(L)^WT^ and HOW(L)^TTAA^. C. Treatment with the MAPKK/MEK inhibitor U0126 decreases HOW phosphorylation. HOW proteins were immunoprecipitated with an anti-HOW antibody from cells transfected with *how(l)^WT^* treated (or not) with U0126, or with *how(l)^TTAA^* and reacted with anti-pTP antibody (top panel) or with anti-HOW antibody (2^nd^ from the top). The crude extract was reacted with anti-pERK antibody (2^nd^ from the bottom), to confirm the extent of U0126 inhibition, and anti-Actin (bottom panel) as a loading control. C′. Quantification of the reduction of phosphorylation following treatment with U0126. For each sample, the ratio between the pTP band measurement and the total HOW band was calculated, and normalized to the HOW(L)^WT^ (non-treated sample) ratio. Results shown are the average of three experiments; error bars indicate SEM. Following U0126 treatment, HOW phosphorylation was reduced to 0.47±0.07 relative to non-treated cells (P = 0.0021, unpaired t-test, n = 3). D. Same as in C, except cells were treated with TPA/PMA. A representative experiment is presented. D′. Quantification of three TPA treatment experiments, in which HOW phosphorylation was increased by 1.28±0.06 (P = 0.0076, unpaired t-test, n = 3).

**Figure 3:**
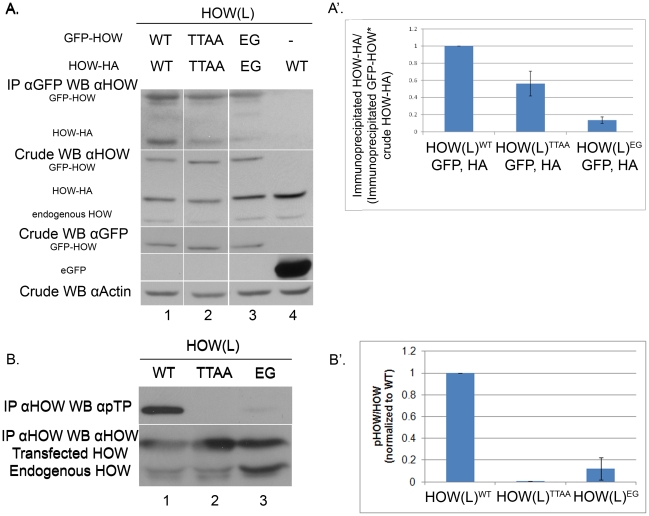
HOW(L) phosphorylation correlates with its homo-dimerization. A. Phospho-mutant HOW(L)^TTAA^ oligomerizes to a lesser extent than HOW(L)^WT^. S2R+ cells were co-transfected with expression vectors for the following: *gfp- how(l)^WT^* and *how(l)^WT^-HA* (1) *gfp- how(l)^TTAA^* with *how(l)^TTAA^-HA* (2); *gfp-how(l)^EG^* with *how(l)^EG^-HA* (3); or *gfp* with *how(l)^WT^-HA* (4) as a control. IP was carried out using anti-GFP antibody and Western blotting was performed with anti-HOW. Crude lysate was reacted with anti-HOW (2^nd^ panel from the top), anti-GFP (to visualize GFP-HOW (lanes 1–3) and the GFP control (lane 4)), as well as with anti-Actin. A′. Quantification of lanes 1–3 from three experiments as described in A. For each sample, the ratio between the Co-immunoprecipitated HA protein and its total crude level was normalized to the amount of the GFP protein that pulled it down. HOW^TTAA^ (2) exhibited partial dimerization (0.56±0.14, P = 0.039 unpaired t-test, n = 3). The HOW^EG^ (3) is almost completely non-dimerized (0.13±0.04, P<0.0001, unpaired t-test, n = 3). B. HOW(L) protein that is unable to homo-dimerize undergoes significantly less phosphorylation. S2R+ cells were transfected with either *how(l)^WT^*, *how(l)^TTAA^* or the *how(l)^EG^* mutant construct. Proteins were immunoprecipitated using anti-HOW antibody. The amount of lysate used for the IP of the HOW(L)^EG^ protein was doubled, in order to obtain a comparable amount of immunoprecipitated HOW protein (since it does not dimerize, less protein precipitates). B′. Quantification of two of several experiments in which the total levels of the different proteins were comparable. For each sample, a ratio between the pTP band measurement and the total HOW band was calculated, and normalized to the HOW(L)^WT^ ratio. Error bars indicate SEM (HOW(L)^EG^ 0.12±0.10).

Next, we tested whether MAPK/ERK could phosphorylate HOW *in vitro*. Briefly, HOW(L)^WT^ and HOW(L)^TTAA^ were transcribed and translated *in vitro* in the presence of S^35^-Methionine, incubated with recombinant activated ERK2, and run on an SDS gel. Incubation of *in vitro* translated Yan, an established MAPK substrate protein [38] with active Erk2 resulted in a protein mobility shift on SDS Page as compared to the unphosphorylated protein (Figure 2A, lanes 1–4). Importantly, HOW(L)^WT^ but not HOW(L)^TTAA^ also displayed a MAPK-dependent mobility shift (lane 5,7), indicating that MAPK phosphorylates HOW and that the phosphorylation event occurs on the predicted residues (Figure 2A, arrow). However, under these conditions, HOW underwent only partial phosphorylation, as a relatively small fraction of HOW was shifted (see below, Figure 3).

### HOW is phosphorylated in *Drosophila* S2R+ cells on MAPK/ERK consensus sites

We next tested whether HOW is phosphorylated on its MAPK/ERK consensus sites using *Drosophila* S2R+ cells. HOW(L)^WT^ and HOW(L)^TTAA^ were expressed in these cells, precipitated with an anti-HOW antibody, and subjected to Western blot analysis using an anti-phospho-Thr-Pro (anti-pTP) antibody (Figure 2B). HOW(L)^WT^ protein reacted with the anti-pTP antibody, whereas the HOW(L)^TTAA^ form did not. This indicated that in unstimulated cells HOW is phosphorylated at least on one TP site.

We have also examined the potential phosphorylation of a shorter HOW isoform, HOW(S), which differs from HOW(L) only at the C terminus [13], . Interestingly, although HOW(S)^WT^ possesses all of the sites predicted to be phosphorylated by, and to bind MAPK, it did not react with the anti-pTP antibody when tested in a similar manner (Figure 2B). This difference may stem from the distinct subcellular localization of the different HOW isoforms; while HOW(L) is nuclear and is thus probably more accessible to active MAPK/ERK, HOW(S) is mostly expressed in the cytoplasm. We therefore limited our analysis to the phosphorylation of HOW(L).

### MAPK/ERK is phosphorylating HOW in S2R+ cells

We further confirmed the identity of the kinase phosphorylating HOW in S2R+ cells by employing the specific MAPKK/MEK inhibitor, U0126 [40] (Figure 2C). Treatment of the cells with U0126 led to a significant decrease in pERK levels (compare the left and middle lanes in Figure 2C). Notably, in the treated cells a marked reduction was observed in the phosphorylation of immunoprecipitated HOW detected by anti-pTP antibody (top panel) in comparison to non-treated cells (phosphorylation was reduced to 0.47±0.07 fold of control, n = 3, Figure 2C′). This result indicates that HOW phosphorylation is dependent on MAPKK/MEK. Interestingly, the phosphorylation of HOW on the TP sites was not completely eliminated, as expected from the effective reduction in pERK levels, suggesting that another kinase might also be involved in the phosphorylation of at least one of the TP sites.

In a complementary approach, we stimulated MAPK/ERK signaling using Phorbol 12-Myristate 13-Acetate (TPA/PMA). This compound is an activator of protein kinase C [41], which in turn leads to activation of MAPK/ERK [42]. Treatment with TPA increased HOW phosphorylation, as indicated by stronger reaction with the pTP antibody (Figure 2D, quantification of 3 experiments is shown in Figure 2D′).

Taken together, these experiments demonstrate that HOW is phosphorylated by MAPK/ERK in *Drosophila* S2R+ cells.

### MAPK/ERK-dependent phosphorylation stabilizes HOW dimers

What could be the reason for the partial phosphorylation of *in vitro* translated HOW by activated ERK (Figure 2A)? We considered the possibility that the incomplete phosphorylation is due to a limited degree of HOW dimerization that occurred under our experimental conditions.

Accordingly, we examined whether MAPK/ERK phosphorylation was related to the degree of HOW dimerization by performing dimerization experiments with HOW^WT^ and HOW^TTAA^. Both HOW variants were fused to GFP and expressed in S2R+ cells together with either HA-tagged HOW^WT^ or HOW^TTAA^, respectively. We immunoprecipitated the GFP-HOW(L) protein using an anti-GFP antibody, and tested, using anti-HOW antibody, the co-precipitation of HOW(L)-HA (Figure 3A). We differentiated between GFP-HOW, HOW-HA and the endogenous HOW by virtue of their different molecular weights. Indeed, HOW^WT^-HA readily co-immunoprecipitated with GFP^WT^-HOW (Figure 3A, lane1), indicating the two proteins oligomerized, presumably as dimers. A negative control protein (eGFP) did not precipitate HOW(L)-HA, demonstrating the specificity of the interaction (Figure 3A, lane 4). Interestingly, the non-phosphorylatable HOW^TTAA^ co-immunoprecipitated less efficiently (lane 2). Quantification of the experiments, while normalizing the co-precipitated HOW-HA levels to its levels in the crude extract as well as to the immunoprecipitated HOW-GFP, showed that co-precipitation of the phospho-mutant HOW was reduced by about 40% compared to that of HOW^WT^ (Figure 3A′). In addition, we attempted to examine the effect of enhanced phosphorylation on HOW dimerization, using a putative phospho-mimicking mutant variant, in which the two Thr residues were replaced by Aspartic acid (HOW(L)^TTDD^). However, this mutation did not appear to mimic phosphorylated HOW, in this as well as in other assays (data not shown). As a control for the dimerization assay, we mutated HOW on a Glutamic acid residue, changing it to Glycine (E106G, HOW^EG^) and similarly tagged it with HA or GFP. This residue was previously shown to be essential for QKI [23] and GLD-1 dimerization [24]. As expected, the amount of co-precipitated HOW^EG^-HA was reduced to about 10% of wild-type levels (Figure 3A lane 3 and Figure 3A′), confirming the reliability of our dimerization assay. Strikingly, testing the phosphorylation state of HOW dimerization mutant HOW^EG^ using anti-pTP antibody revealed that this HOW mutant essentially does not undergo phosphorylation by MAPK/ERK (Figure 3B lane 3, 3B′).

Collectively, the above experiments strongly suggest that MAPK-dependent phosphorylation occurs only on HOW dimers, although we cannot exclude a possible change of conformation in the monomeric structure of HOW^EG^ [24]. Our results also further support the idea that phosphorylation stabilizes HOW dimerization since the extent of dimer formation of HOW^TTAA^ is significantly reduced.

### Phosphorylation of HOW strengthens its binding to RNA

**Figure 4:**
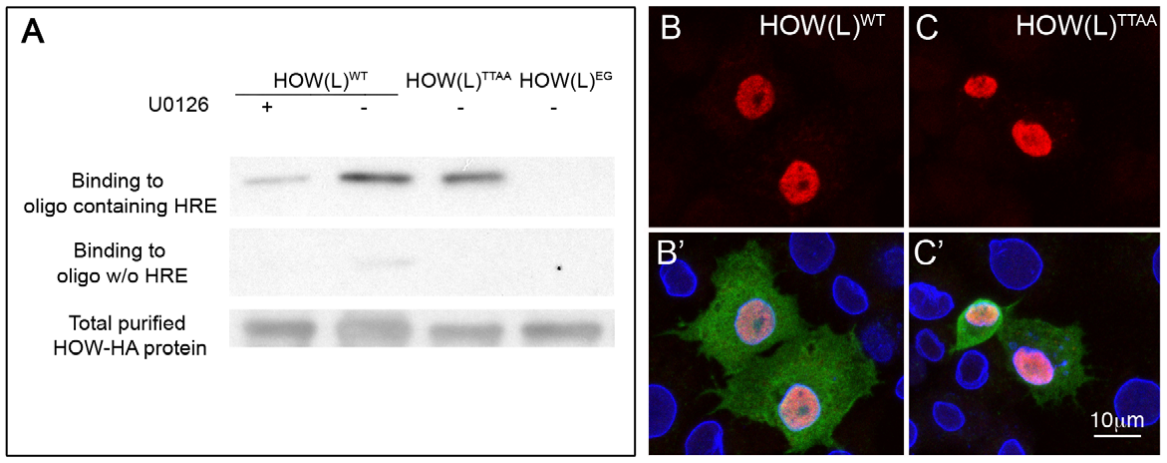
HOW phosphorylation strengthens its affinity to RNA, while not affecting its subcellular localization. A. *In vitro* RNA-protein binding assay. HA tagged HOW(L)^WT^, HOW(L)^TTAA^ or HOW(L)^EG^ constructs were purified from S2R+ cells by immunoprecipitation of the HA tag, followed by elution with free HA peptide. HOW(L)^WT^ transfected cells were either treated with U0126 or not treated. Equal volumes of purified proteins were incubated with biotin labeled 12 nt oligomers that either bind HOW (top panel) or do not bind HOW (middle panel). The complexes were incubated with avidin beads, eluted by boiling in sample buffer and reacted with anti HOW in a Western blot. The amounts of HOW protein used for each reaction were comparable (bottom panel). B–C′. Localization of HOW(L) was not altered due to the mutations in the Thr residues. Cells expressing either HOW(L)^WT^ (B,B′) or HOW(L)^TTAA^ (C,C′) were stained for HOW (red, B–C). Merge images (B′–C′) also present GFP (green, marks transfected cells) and Lamin (blue).

To address whether the phosphorylation of HOW influences its ability to bind RNA, we performed an RNA binding assay. In this experiment, wild-type HA-tagged HOW(L) was affinity purified from S2R+ cells that were either grown in normal medium (in which HOW is phosphorylated, see Figure 2C) or in medium containing the MEK inhibitor U0126 (in which HOW phosphorylation is reduced, shown in Figure 2C). The RNA-binding activity of purified HOW-HA was then tested using biotin-labeled RNA oligomers that either contained or did not contain the HOW response element (HRE) [43]. A significant reduction of about 70% in the binding of HOW to the RNA was detected following treatment with the MEK inhibitor U0126 (Figure 4A, left lane). In addition, HOW(L)^EG^, the mutant form that does not dimerize, exhibited an extremely low RNA binding activity, supporting a role for the dimerization of HOW in RNA binding (Figure 4A, right lane). Notably, the non-phosphorylatable HOW(L)^TTAA^ only showed slightly reduced binding (Figure 4A, second lane from the right, about 20% reduction), possibly due to its ability to dimerize with endogenous, phosphorylated HOW protein (contrary to the U0126-treated cells, where all HOW proteins are less phosphorylated). This experiment demonstrates that ERK-dependent phosphorylation of HOW enhances not only its homodimerization but also its RNA binding activity, suggesting that both functions are linked.

### Thr phosphorylation does not alter the subcellular localization of HOW

Phosphorylation alters the subcellular localization of Sam68 [4], [32], raising the possibility that, via this mode of regulation, MAPK impinges on HOW's ability to bind and regulate its RNA targets. To address this issue, we transfected S2R+ cells with HOW^WT^ or HOW^TTAA^ tagged with HA, and stained with an anti-HA antibody. We did not detect any alteration in the subcellular localization of HOW(L)^TTAA^ relative to HOW(L)^WT^ (Figure 4B–4C′). Thus, HOW Thr phosphorylation does not alter its subcellular localization.

### HOW is phosphorylated on T64 in the embryo *in vivo*

**Figure 5:**
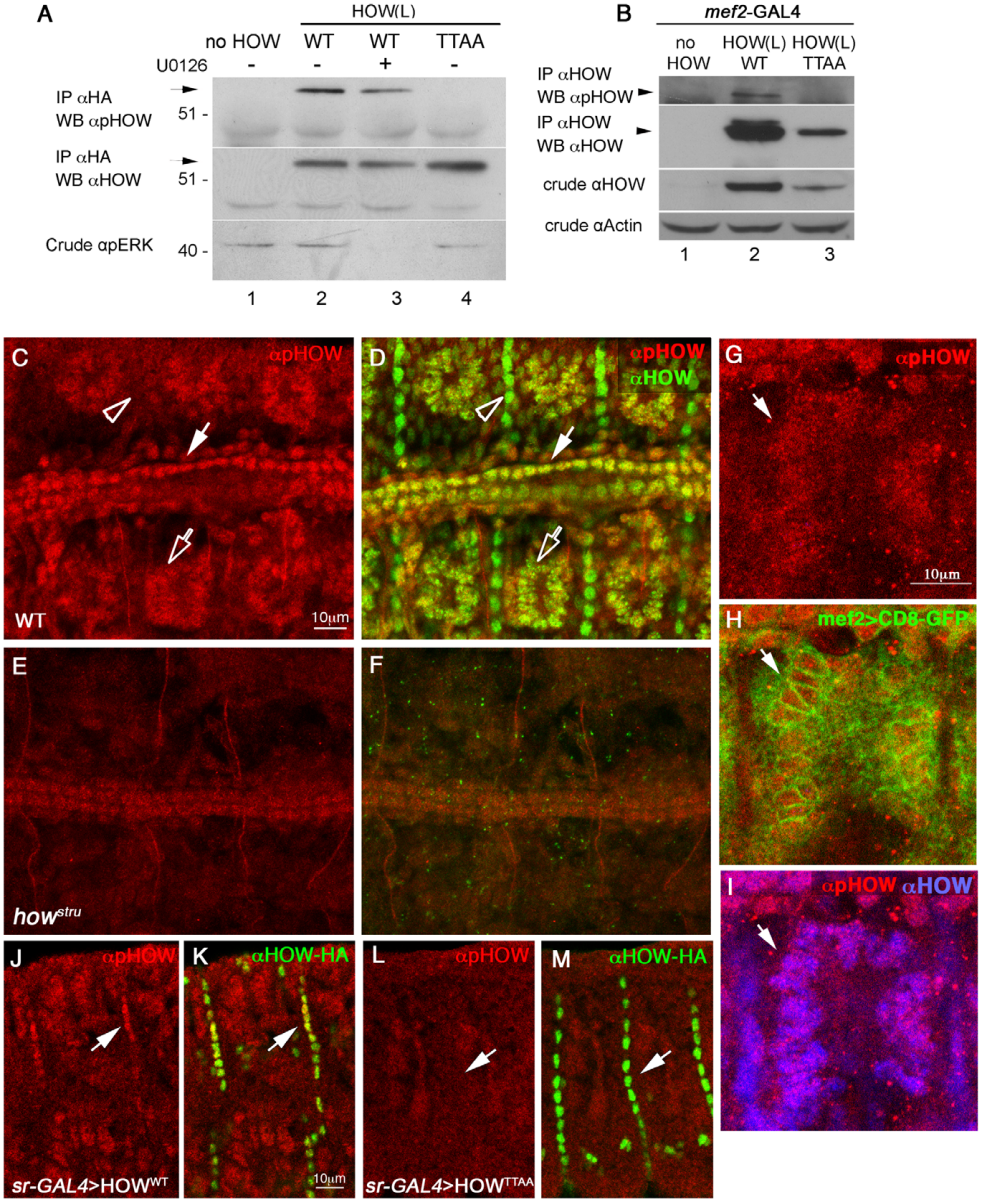
Phosphorylated HOW is detected in the nuclei of somatic muscles and cardioblasts. A. Anti-pHOW (pT64) antibody reacts with HOW, in a phosphorylation-dependent manner. HOW protein was immunoprecipitated with an anti-HOW antibody from S2R+ cells transfected with either HOW(L)^WT^, treated (or not) with U0126 (lanes 3, 2 respectively), with HOW(L)^TTAA^ (lane 4), or no HOW (lane 1). The IP was reacted with anti-pHOW (pT64) antibody (top) or with anti-HOW antibody (middle). The crude extract was reacted with anti-pERK antibody (bottom), confirming U0126 inhibition. Note specific reactivity of the anti-pHOW antibody. B. Anti-pHOW (pT64) antibody reacts with HOW protein overexpressed in embryonic muscles and heart. Transgenic UAS-HOW(L)^WT^ (lane 2) and UAS-HOW(L)^TTAA^ (lane 3) flies or wild-type controls (lane 1) were crossed to the *mef2*-GAL4 driver line. Embryos were collected, and protein extracts were subjected to IP with an anti-HA antibody followed by Western with anti-pHOW (pT64) antibody (top), and anti-HOW antibody (2^nd^ from top). Crude extracts were reacted with anti-HOW (2^nd^ from bottom), and anti-Actin (bottom) as a loading control. C-F. *Drosophila* embryos stained with anti-pHOW (pT64) exhibit specific staining in somatic muscles and heart cardioblasts. Stage 16 WT (C,D) and *how ^stru^* mutant (E,F) embryos were stained with anti-pHOW (pT64) (red, C–F) and anti-HOW (green in merge D,F). Embryos are oriented dorsal to the top and anterior to the left. (C,D) Note the staining in muscles (hollow arrow), heart cardioblasts (white arrow) and the lack of pHOW staining in the muscle attachment sites (arrowhead). (E,F) Weak staining in cardioblasts in *how ^stru−/−^* mutants. G–I. *Drosophila* embryos expressing CD8-GFP, which localizes at the plasma membrane, but also often concentrates in the ER surrounding the nuclei, in muscles under *mef2*-GAL4, were stained with anti-pHOW (red, G) shown merged with GFP (green, H) and with HOW (blue, I). J–M. HOW(L) overexpressed in embryonic tendon cells reacts with the anti-pHOW (pT64) antibody. Embryos expressing UAS-HOW(L)^WT^-HA (J,K) or UAS-HOW(L)^TTAA^–HA (L,M) in the tendon cells under *sr*–GAL4 were stained with anti-pHOW (pT64) (red, J–M) and anti-HA (green, K, M, merged with the anti-pHOW staining). Arrows mark tendon cells reactive with anti-pHOW upon expression of HOW(L)^WT^ (J,K), or non-reactive upon expression of the phospho-mutant (L,M).

To follow the pattern of HOW phosphorylation *in vivo*, we generated a polyclonal antibody designed to specifically recognize HOW only when it is phosphorylated on the more conserved Thr residue, T64 (we refer to the antibody as anti-pHOW (pT64) (see Materials and Methods)). We first confirmed the specificity of the antibody by transfecting S2R+ cells with *how(l)^WT^*, *how(l)^TTAA^*, and by treating a sample of the HOW(L)^WT^ expressing cells with the MAPKK/MEK inhibitor U0126. We performed immunoprecipitation (IP) with an anti-HOW antibody and used anti-pHOW (pT64) antibodies in Western blot analysis (Figure 5A). The antibody reacted with the immunoprecipitated HOW(L)^WT^ (lane 2) but not with HOW(L)^TTAA^ (lane 4) nor with non-transfected cells (lane 1). In the sample treated with U0126 (lane 3), the reactivity of the antibody was significantly reduced, suggesting the antibody indeed detects HOW phosphorylation, particularly on T64.

We used the anti-pHOW (pT64) antibody to identify tissues in which HOW is phosphorylated (Figure 5C). To this end, we stained wild-type *Drosophila* embryos, employing *how* mutant (*how^stru−/−^*) embryos, in which zygotic HOW is not produced, as a control for antibody specificity. The embryos were also co-stained with a general anti-HOW antibody. Specific anti-pHOW staining was detected in the nuclei of somatic muscles (Figure 5C, open arrow) as well as in the heart cardioblasts (Figure 5C, white arrow). This staining co-localized with anti-HOW staining (Figure 5D, corresponding arrows) and was reduced to background levels in *how^stru^* mutants (Figure 5E, 5F). Importantly, phosphorylated HOW was confined to the nuclei of the somatic muscles that were marked with muscle-specific expression of CD8-GFP (which localizes to the cell membrane but often concentrates in the ER surrounding the nuclei), as shown in Figure 5G–5I. This result indicates that HOW is phosphorylated on T64 in muscle nuclei.

Intriguingly, the anti-pHOW (pT64) antibody did not label tendon cells, which exhibited a strong anti-HOW staining (Figure 5C, 5D, open arrowheads), further validating the specificity of our antibody. The lack of pHOW staining is not simply due to low levels of MAPK in these cells, since previous analysis demonstrated high level of phospho-ERK in tendon cells at this stage [44]. We therefore hypothesized that the HOW(L) isoform found to be specifically phosphorylated by MAPK/ERK (Figure 2) is not expressed at high levels in tendon cells at this developmental stage and that the anti-HOW staining is mainly detecting HOW(S), which does not undergo phosphorylation in S2R+ cells (Figure 2B). To directly address this possibility, we generated transgenic flies expressing HA-tagged versions of HOW(L)^WT^ or HOW(L)^TTAA^ under UAS-GAL4 binding sequences. The expression of HOW(L)^WT^ or HOW(L)^TTAA^ was driven in embryonic tendon cells by the tendon-specific driver, *sr*-GAL4, and the embryos were stained for pHOW (Figure 5J–5M) Whereas over expression of HOW(L)^WT^ in tendon cells led to positive nuclear staining with the anti-pHOW antibody (Figure 5J, 5K arrows), overexpression of HOW(L)^TTAA^ did not result in such a staining (Figure 5L, 5M arrows). This experiment shows that the kinase required for HOW phosphorylation on T64 is activated in tendon cells, and that the lack of reactivity of the antibody in wild-type tendon cells at late embryonic stages is due to low levels of HOW(L) in this tissue. This is also consistent with the cytoplasmic HOW staining characteristic of the HOW(S) isoform present at stage 16 embryos [39].

Based on these results, we conclude that HOW(L) is phosphorylated on T64 in the nuclei of somatic muscles and heart cardioblasts. To further verify that HOW undergoes Thr phosphorylation in embryonic somatic muscles, we also expressed HA–tagged HOW(L)^WT^ or HOW(L)^TTAA^ in muscles using the *mef2*-GAL4 driver. The HOW variants were subsequently immunoprecipitated using anti-HA conjugated beads and reacted with anti-pHOW (pT64) antibodies on Western blots (Figure 5B, lane 2). Only HOW(L)^WT^ but not HOW(L)^TTAA^ (lane 3), was detectable by the anti-pHOW antibody. In addition, an anti-HOW antibody identified an additional upper band that might represent phosphorylated HOW (lane 2). To conclude, these findings strongly indicate that HOW(L) undergoes phosphorylation on T64 in embryonic somatic and cardiac muscles and that phosphorylated HOW in muscles is localized specifically to the nucleus.

### HOW(L) downregulates Sallimus in muscles in a phosphorylation-dependent manner

To further characterize the physiological significance of MAPK/ERK-dependent phosphorylation of HOW, we focused on somatic muscles, a tissue in which a high degree of HOW phosphorylation was detected. Recently, HOW was shown to be essential for muscle sarcomerization [45]. Specifically, in larvae where *how* was knocked down (using *how* RNAi expressed specifically in muscles), the sarcomeric organization is aberrant. In these larvae, the Z discs appear discontinuous and spotty, a phenotype similar to that caused by the knock down of several genes encoding sarcomeric proteins [45]. To address the possibility that this phenotypic resemblance is a result of regulation of one or more of these proteins by HOW, we assessed the levels of different sarcomeric proteins in 3^rd^ instar larvae, in which we reduced HOW levels using RNAi mediated knock-down in muscles. To enhance the effect of *how* down-regulation, the larvae used were also heterozygous for *how^stru^*.

**Figure 6:**
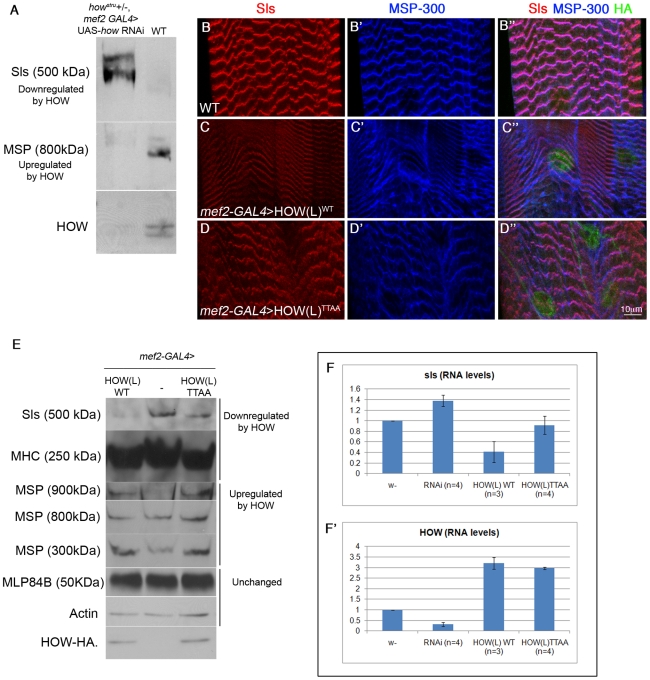
HOW regulates the levels of Sls RNA and protein in a phosphorylation-dependent manner. A. Sls and MSP-300 are directly or indirectly regulated by HOW. Extracts from larvae in which HOW was down-regulated by expressing *how* RNAi (aimed against all HOW isoforms) under *mef2*-GAL4, on the background of *how^stru^* heterozygotes, were reacted with anti-Sls, MSP-300, and HOW antibodies and compared to larvae heterozygotes for *mef2*-GAL4 in Western blot analysis. Down-regulation of HOW (lower panel) led to upregulation of Sls (upper panel) and down-regulation of MSP-300 (middle panel). B–D. Overexpression of HOW leads to reduction of Sls protein levels, in a phosphorylation-dependent manner. Body wall muscles from 3rd instar larvae expressing *mef2*-GAL4 alone (B,B′,B″), UAS-HOW(L)^WT^-HA, or UAS-HOW(L)^TTAA^-HA driven by the *mef2*-GAL4 driver (C,C′,C″ and D,D′,D″, respectively) were stained with anti-Sls (B,C,D - red), anti-MSP-300 (B′,C′,D′- blue) and anti-HA (B″,C″,D″ - green, shown within merge). Quantification of at least three larvae from each genotype showed that HOW(L)^WT^ over-expression reduced Sls intensity to 0.71±0.05 of the intensity of the *mef2* -GAL4 heterozygotes, while the HOW(L)^TTAA^ protein only reduced it to 0.83±0.03 of control (P = 0.049, ANOVA test). E. Overexpression of HOW(L) in larval muscles alters the levels of multiple muscle proteins, of which only Sls is affected in a HOW phosphorylation-dependent manner. Protein extracts from 2^nd^ instar larvae expressing *mef2*-GAL4 alone (middle), or either HOW(L)^WT^ (left) or HOW(L)^TTAA^ (right) driven by *mef2*-GAL4 were analyzed by Western blotting with the following antibodies (from top to bottom): anti-Sls, anti-MHC, anti-MSP300 (reacts with three different isoforms), anti-MLP84B, anti-Actin, and anti-HOW. Note the comparable levels of HOW(L)^WT^ and HOW(L)^TTAA^ expression, and the relatively mild reduction in Sls when HOW(L)^TTAA^ is overexpressed. F–F′. HOW regulates Sls at the RNA level. RNA was extracted from single 3^rd^ instar larvae, and real-time PCR was performed with *sls* primers (F) and *how* primers (F′), both normalized to *rp49* as a control. From left to right: *mef2*-GAL4 heterozygotes (w−), *how^stru+/−^*, *mef2*>*how* RNAi (RNAi), and *mef2*-GAL4 driving either HOW(L)^WT^ or HOW(L)^TTAA^. Error bars indicate SEM. While reduction of HOW levels elevated *sls* (1.38±0.11, P = 0.039), overexpression of HOW(L)^WT^ reduced it more efficiently (0.54±0.20, P = 0.08) than HOW(L)^TTAA^ (0.92±0.18, P = 0.78) (Student's t-test, n = 3 or 4).

Under these conditions, we observed an increase in the 500 kDa isoform of Sls, a giant protein that serves as a scaffold in the sarcomere, linking the Z-discs to the thick filaments [33] (Figure 6A). In contrast, the levels of MSP-300 (isoform 800 kDa), a Z-disc protein [46], [47], were decreased (Figure 6A). This suggests that in wild-type larval muscles, Sls is down-regulated by HOW, while MSP-300 is elevated. Although these results do not distinguish between direct and indirect effects, nonetheless, we used the levels of these two proteins in muscles as readout of the activity of phosphorylated (HOW^WT^) versus non-phosphorylated HOW (HOW^TTAA^) in this developmental process.

Accordingly, we expressed comparable amounts of either HOW^WT^ or HOW^TTAA^ in muscles (using the *mef2*-GAL4 driver) and followed the expression of Sls and MSP-300 by fluorescent labeling (Figure 6B–6D) or by Western blot analysis (Figure 6E) in 3^rd^ instar larvae. As expected, over-expression of HOW(L)^WT^ resulted in reduced levels of Sls (Figure 6C). This was confirmed by measuring the immunofluorescence intensity of Sls in Z-discs (see legend to Figure 6). Importantly, overexpression of HOW(L)^TTAA^ exhibited a significantly milder effect on Sls levels (Figure 6D). In contrast, over-expression of either HOW^WT^ or HOW^TTAA^ resulted in a similar aberrant distribution of MSP-300 (Figure 6C′, 6D′).

We also quantified the changes in protein levels by employing Western blot analysis of extracts from the body walls of several larvae, expressing driver alone (*mef2*-GAL4), or together with HOW^WT^ or HOW^TTAA^ (Figure 6E). Consistent with the immunofluorescent staining, the anti-Sls antibody reacted with a major band of 500 kDa that was downregulated following HOW(L)^WT^ overexpression. A much milder effect was observed following HOW(L)^TTAA^ overexpression (Figure 6E, upper band). Myosin Heavy Chain (MHC) [48], [49] was reduced following expression of both HOW^WT^ and HOW^TTAA^ (Figure 6E second band from top). Of the three distinct bands representing MSP-300 (900 kDa, 800 kDa, 300 kDa), the 900 kDa and the 300 kDa bands were elevated, whereas that of 800 kDa was unaffected following HOW^WT^ overexpression. No significant differences were found between the effect of HOW^WT^ and HOW^TTAA^ on all three bands. The levels of two additional muscle proteins, MLP84B [46] and Actin remained unchanged in these genetic backgrounds.

To conclude, the down-regulation of the newly-identified HOW target, Sls, is dependent on the phosphorylation state of HOW, whereas MSP-300 and MHC are regulated by HOW in a phosphorylation-independent manner. This discrepancy may be due to differential activities of HOW (e.g. regulation at the level of RNA degradation versus alternative splicing).

Given that HOW is an RBP, we next sought to determine whether HOW controls *sls* RNA levels, and if so, whether phosphorylation is important for this type of regulation. To this end, we purified total RNA from the body walls of single 3^rd^ instar larvae and performed real-time PCR to quantify the levels of the *sls* and *how* transcripts (*rp49* RNA served as control) using the SYBR green method. We compared larvae from a wild-type background (w^−^), to larvae heterozygous for *how^stru^* which expressed *how* RNAi in muscles, and to larvae over-expressing either HOW(L)^WT^ or HOW(L)^TTAA^ in muscles. In larvae expressing lower levels of *how* (Figure 6F′, second left bar, RNAi), *sls* mRNA was upregulated (Figure 6F). Importantly, HOW(L)^WT^ was more efficient in the down-regulation of *sls* RNA relative to HOW(L)^TTAA^ (Figure 6F right bars). Although this result was not statistically significant, the trend seen at the level of RNA is consistent with the results obtained in the protein analysis. Thus, our data demonstrate that HOW(L) has a role in the down-regulation of *sls* mRNA and protein levels in muscles, and that this process is dependent on HOW phosphorylation.

### Downregulation of MAPK signaling in larval muscles results in diminished phosphorylation of HOW and elevation of Sallimus protein levels

**Figure 7:**
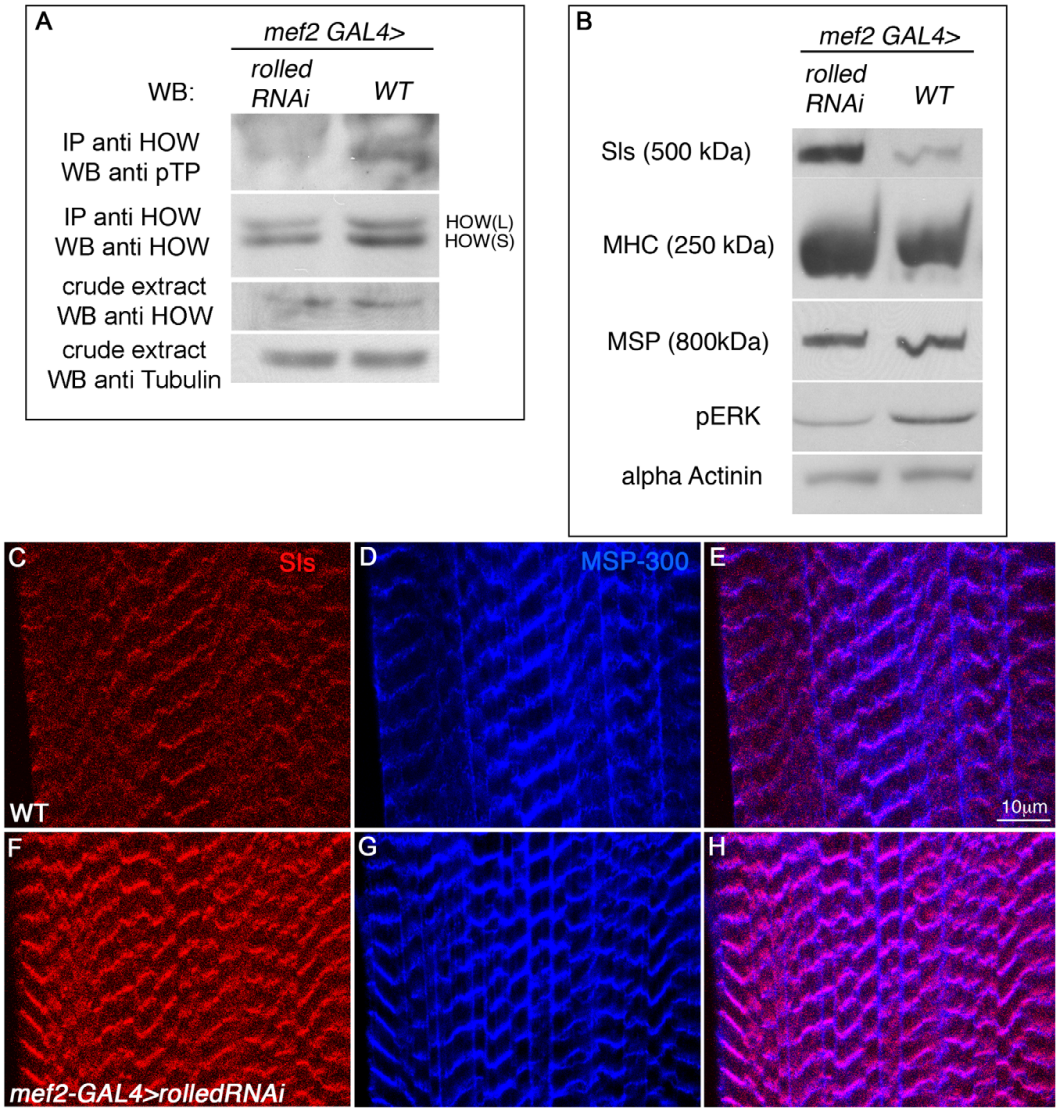
*Drosophila* MAPK *rolled* is directing HOW phosphorylation in muscles and regulation of Sallimus levels. A. HOW was immunoprecipitated from lysates of 3^rd^ instar larvae either expressing *rolled* RNAi in muscles under *mef2*-GAL4 (left) or of wild-type *mef2*-GAL4 heterozygotes (w−, right) and reacted with a pTP antibody to observe its phosphorylation level and with anti-HOW to compare total HOW levels. The crude extract was reacted with anti-HOW and anti-Tubulin as a loading control, showing that the total quantity of HOW is not significantly altered by MAPK/Rolled levels. Note the reduction in HOW phosphorylation following expression of *rolled* RNAi. B. Reduction in MAPK/Rolled levels elevates Sls protein levels. Same genotypes as in A were analyzed by Western with the following antibodies (from top to bottom): anti-Sls, anti-MHC, anti-MSP300, anti-pERK (to verify the reduction in activated MAPK in these larvae) and anti-α-Actinin as a loading control. C–H. Wild-type (C–E) and *mef2*-GAL4>*rolled* RNAi (F–H) larvae were stained with anti-Sls antibody (red, C,F) and anti MSP-300 (blue, D,G). Merge is shown in E,H. Note the significant elevation in Sls levels following down regulation of MAPK/Rolled levels.

We next examined whether HOW phosphorylation in muscles, and its resulting elevated activity in the regulation of Sls levels (Figure 6), are indeed dependent on MAPK signaling. To test this idea, we expressed RNAi for the *Drosophila* MAPK gene *rolled* in larval muscles under *mef2*-GAL4 regulation, immunoprecipitated HOW from protein extracts of these larvae and examined HOW phosphorylation using Western blot analysis with an anti-pTP antibody (Figure 7A). Importantly, in the larvae where *rolled* was down regulated (note the reduction in the levels of pERK in Figure 7B), we observed diminished phosphorylation of HOW, compared to wild-type larvae (Figure 7A). The phosphorylation of HOW in wild-type larvae was present in a band corresponding to the HOW(L) protein. Thus, phosphorylation of HOW(L) in larval muscles *in vivo* is dependent on MAPK signaling, as in S2R+ cells and in an *in vitro* kinase assay (Figure 2).

Since *rolled* down regulation resulted in decreased HOW(L) phosphorylation, we hypothesized that HOW(L) activity will be lowered in these larvae, resulting in elevated levels of Sls. Indeed, using a Western blot analysis (Figure 7B) we find that in larvae expressing *rolled* RNAi under *mef2*-GAL4, Sls levels are significantly elevated. The increase in Sls levels is specific, as only a mild increase is observed in MHC levels, while MSP and α-Actinin levels are unaffected (Figure 7B). The marked elevation of Sls levels following reduction of *rolled* in the larval muscles is also readily detectable in individual muscles (Figure 7C, 7F), while MSP-300 levels remain constant (Figure 7D, 7G, merge in Figure 7E, 7H). Thus, we conclude that in wild-type larval muscles, MAPK signaling is required for HOW(L) phosphorylation and reduction of Sls protein levels.

## Discussion

STAR proteins regulate tissue differentiation in a wide range of species including nematodes [50], flies [17], Zebrafish [8], mice, and humans [51]. They function by controlling diverse posttranscriptional events, often forming specific protein-RNA complexes mediated by 3′UTR sequences of their target mRNAs, or with alternatively spliced introns [2]. In this study we reveal a molecular mechanism regulating the activity of the STAR protein HOW. Our findings demonstrate that HOW is phosphorylated on Thr residues embedded within conserved MAPK consensus sequences, both in cultured cells as well as in muscle cells and heart cardioblasts (Figure 2, 5), and that this phosphorylation is executed by MAPK/ERK (Figure 2, 7). Significantly, our results provide novel molecular insights to the importance of this phosphorylation, demonstrating that phosphorylation stabilizes HOW dimer formation (Figure 3). Moreover, because phosphorylation presumably occurs only on dimers, and phospho-dimers are more stable, we propose that a feed-forward loop ensures that a large fraction of HOW dimers are formed following a short temporal burst of MAPK/ERK activation. Importantly, since STAR proteins are evolutionarily conserved, the novel mode of regulation that we have uncovered might have important implications for other members of the QKI sub-family.

Two of the four potential MAPK/ERK docking sites reside within the HOW QUA1 domain. The close proximity between these sites, the MAPK/ERK phosphorylation sites, and the E106 residue critical for dimerization ([23], [24], Figure 3), all of which are highly evolutionarily conserved (Figure 1), is consistent with local conformational changes in the QUA1 domain induced by phosphorylation, which further lead to dimer formation and stabilization. HOW dimerization might be essential for a number of its characteristics; first, it enables binding to several HREs present on a single target RNA, leading to an overall higher affinity of HOW to its target RNA. For example, the 3′UTR of the HOW target *stripe* contains three consecutive HREs and a half site that resides between the first and second HREs. We have previously shown that a higher affinity of HOW is observed when multiple HREs are clustered together [43]. HOW dimerization and binding to two sites may also contribute to the formation of a secondary RNA structure, thus facilitating RNA processing. Interestingly, HOW dimerization apparently enhances its RNA binding ability even to a single binding site (Figure 4A).

Additionally, HOW dimerization might also potentiate the recruitment of other proteins/enzymes to the vicinity of the targeted mRNA. This may occur either if one subunit associates with the target RNA, and the other with a specific enzyme that induces modification/degradation of the target RNA, or if the formation of HOW dimers enables recruitment of proteins that would not associate with its monomeric form. A similar mechanism has been proposed to take place when GLD-1 dimers associate with their RNA target(s) close to the polyadenylation site, possibly recruiting an E3 ubiquitin ligase complex to this site. Ubiquitination of the polyadenylation complex would subsequently lead to shortening of the poly-A tail [24]. A mechanism for HOW activity that includes protein interactions with binding partners has yet to be described, but such an interaction is highly likely, since HOW does not contain any recognizable catalytic domains that could independently lead to mRNA degradation.

We show that a HOW mutant unable to form dimers exhibits a greatly diminished RNA binding capacity (Figure 4A, HOW^EG^ mutant). This is also in line with our findings that HOW binding to RNA is significantly reduced when it is hypophosphorylated, and that phosphorylated HOW has a higher tendency to form dimers. We conclude that dimeric HOW binds RNA with higher affinity than monomeric HOW. This conclusion is in line with the observations that GLD-1 lacking its QUA1 domain has a lower affinity to RNA [26], and possibly applies to other STAR proteins.

To our knowledge, this is the first example of a positive effect of phosphorylation of a STAR protein on its RNA binding capacity. Phosphorylation of Sam68 by MAPK/ERK was demonstrated to have a subtle negative influence on its RNA binding [32], [52], while Tyrosine phosphorylation was shown to more severely impair RNA binding of both QKI [29] and Sam68 [53]. As the Tyrosine residues in HOW are highly conserved with those of QKI, it is highly likely that they are also phosphorylated (Kirenberg and Volk, unpublished data), and that this phosphorylation may have an opposite, negative effect on the ability of HOW to bind its targets. Hence, it is interesting to note that changes in cellular signaling may have the capacity to fine-tune, both positively and negatively, the ability of an RNA binding protein to bind its targets, thus modulating the levels of a variety of mRNAs.

A requirement for HOW in developing muscles had been demonstrated previously [45], but its target RNAs in this tissue have not been characterized. In this study, we identify three different muscle proteins, Sls, MSP-300 and MHC, whose levels are altered by the expression of HOW in this developmental setting. We still do not know whether the mRNAs of these proteins are all directly bound by HOW, and at what level the regulation by HOW occurs, i.e. via control of specific alternative splicing or by regulation of overall mRNA levels. Since *sls* mRNA levels respond to both HOW overexpression and knock-down, and given that the *sls* transcript contains several potential binding sites for HOW (both at the 3′UTR as well as within several introns; not shown), it likely represents a true direct RNA target of HOW.

Even though Sls is a structural protein, its fast turnover in sarcomeres might be essential for maintenance of the sarcomeric architecture [54]. To fulfill this requirement, its protein and RNA half-life should be short. Thus, HOW might play an essential role in promoting destabilization of *sls* mRNA to promote fast exchange of newly formed Sls protein at the Z-disc. Indeed, in muscles where MAPK signaling was downregulated using *rolled* RNAi, a significant elevation in Sls levels is clearly evident (Figure 7). This result is in line with tight regulation of Sls levels occurring in wild-type larvae.

Although the precise RTK signaling pathway that regulates HOW phosphorylation in muscles is yet to be elucidated, the FGFR Heartless, which is expressed by muscle cells throughout their development [55], [56], is an attractive candidate. It is possible that continuous FGFR activation in muscles promotes HOW phosphorylation by MAPK, rendering it more active in controlling the levels of its target mRNAs in this tissue.

In summary, in this study we have unraveled and characterized a novel molecular mechanism at the basis of the activity of the STAR protein, HOW. By linking its activity to MAPK/ERK-dependent phosphorylation and regulation, we provide a mechanistic linkage between HOW phosphorylation, the degree of its dimerization and its biological activity/function. We propose that this mechanism may apply to other STAR proteins, in which the dimerization domain and phosphorylation sites are evolutionarily conserved.

## Materials and Methods

### Expression constructs for cell lines and flies

Mutant HOW(L) constructs (^TTAA^, ^EG^) were created by site directed mutagenesis (Stratagene), following the manufacturer's protocols. pUAST-HOW(L) constructs tagged with HA or fused with GFP were generated in the pTWH and pTGW *Drosophila* Gateway vectors, respectively (T. Murphy, Carnegie Institution of Washington) using the Gateway cloning system (Invitrogen).

### Fly strains

Fly stocks used in this study include w^−^, *mef2*-GAL4 (Bloomington stock center), *mef2*-GAL4, UAS-CD8 GFP (F. Schnorrer, Martinsried, Germany), *stripe*-GAL4 (G. Morata, Madrid, Spain), *how^stru^* [57], *how* RNAi line (ds-HOW) [43], *rolled* RNAi (TRiP HMS00173, Bloomington stock 34855). *UAS-how(l)^WT^-3HA* and *^TTAA^-3HA* were injected to flies by Genetic Services, Sudbury, MA, USA.

### Antibodies

Primary antibodies used in this study include mouse anti-phospho-Thr-Pro (p-Thr-Pro-101) (Cell Signaling Technology), rabbit and rat anti-HOW [39], guinea pig anti-MSP-300 [47], rat anti-Sls (Kettin) (Klg16, MAC155, Abcam), rat anti α-Actinin (Abcam), rabbit anti-MHC (P. Fisher, Stony Brook, NY), mouse anti-Actin (Sigma), rabbit anti-Mlp84bB [58], mouse anti-GFP (Roche), mouse anti-HA (Roche), chick anti-HA (Aves Labs), mouse anti-Lamin ([59], gift from Y. Gruenbaum, Hebrew University), mouse anti-pERK (gift from B. Shilo, Weizmann Institute). The polyclonal anti-pHOW (pT64) antibody was raised in rats against the phospho-peptide PQHL(p)TPQQ (generated by Sigma), corresponding to amino acids 60–67 of HOW. Immunizations were performed by the antibody unit at the Weizmann Institute. The serum was cleaned on beads conjugated to a similar peptide, PQHLQPQQ, in an attempt to reduce background staining that was suspected to be due to another protein containing this sequence. Secondary antibodies used in this study include various Cy3, Cy2, Cy5 and HRP-conjugated antibodies (Jackson ImmunoResearch Laboratories, USA).

### Tissue culture and transfection

S2R+ cells were grown and transfected essentially as previously described [43], except that cells were usually collected for analysis 36 hours after transfection. For inhibition of MAPK/ERK activity, 10 µM U0126 (Sigma) in DMSO (or only DMSO for controls) was administered with the serum-containing media, about 18 h after the transfection. Since U0126 led to a decrease in the efficiency of recovery from transfection, the control cells were transfected with smaller amounts of *how* DNA. Phorbol 12-Myristate 13-Acetate (TPA/PMA) (Sigma) treatment (10 µM in DMSO, or only DMSO for controls) was performed on starved cells, 20 h after transfection, for 15 min.

### Cell lysis

Cells were scraped off the flasks and collected in PBS, washed twice and lysed in 1% NP40 lysis buffer (50 mM Tris pH 7.5, 1% NP40, 100 mM NaCl, 1% Protease inhibitor cocktail (P8340, Sigma), 0.5% Phosphatase Inhibitor Cocktail 1(P2850, Sigma), 20 mM β -glycerol phosphate).

Embryos and larvae were collected and crushed in RIPA buffer (1% sodium deoxycholate, 1% Triton X-100, 0.1% SDS, 0.15M NaCl, 0.05M Tris pH 7.0, supplemented with the same inhibitors).

### Western blotting and immunoprecipitation (IP)

For IP, equal amounts of protein lysate were incubated with protein A/G beads (SC-2003, Santa Cruz) coupled with either rabbit anti-HOW polyclonal antibody or mouse anti-GFP monoclonal antibody, or agarose HA conjugated beads (A2095, Sigma) for 1–2 h at 4°C. Beads were washed three times with the NP40 lysis buffer, and boiled in protein sample buffer to elute the proteins.

MAPK/ERK *in vitro* phosphorylation assay was performed as described [60]. High molecular weight proteins were analyzed by SDS–PAGE using 2.5% acrylamide gels strengthened with 1.5% agarose, essentially as in [61].

### Fixation and immunostaining

Fixation and staining of embryos were done following standard procedures. Larval Flat Preparations were performed essentially as described [62], except the fixation was done in 4% PFA, the primary antibody staining was carried out for 2 h and the secondary for 1 h. Quantification of average intensity of larval muscles images were performed using MATLAB.

S2R+ cells were seeded on Ibidi u-Slide 8 well (0.2*10^6^ cells per well), fixed with 3% PFA for 5 min, permeabilized using 3% PFA+0.1% TritonX-100, stained with primary antibody (1∶200) for 30 min, and secondary antibody (1∶400) for 30 min.

Visualization was carried out using a Zeiss LSM710 confocal system.

### RT–PCR analysis of RNA extracts

Single larvae were flipped over and their interior was cleaned, leaving the carcass only. RNA was extracted by the Nucleospin Purification Kit (Macherey-Nagel) according to the manufacturer's instructions. Equal amounts were used as a template for cDNA preparation using the Verso cDNA kit (Thermo Scientific). Real time PCR was carried out using Fast SYBR Green Mastermix (Applied Biosystems) in a StepOne plus machine (Applied Biosystems). The following primers were used:

*sls* (TGCCCATGCCGAAGACA) and (TGTCTTGTTTGCTGTTACGTTTACAG),

*rp49* (GACCATCCGCCCAGCATAC) and (CCATTTGTGCGACAGCTTAGC),

*how* (AACTTTGTCGGTCGCATTTT) and (CGTCCTCCTTCTTCTTGTCG).

### RNA–protein binding

*In vitro* RNA–protein binding assay was performed essentially as described [43], only that the proteins were not *in vitro* translated, but were purified from S2R+ cells using immunoprecipitation with HA conjugated beads, followed by elution with an HA peptide (Sigma, I2149).

### Software

Phosphorylation sites were predicted by the GPS2.1 program [63]. Protein scheme was generated by DOG2.0 software [64]. Student's t-tests were performed using GraphPad software.
